# Studying the electronic properties of SiO_2_/GO/Pb_3_O_4_/Bi_2_O_3_ composite structure

**DOI:** 10.1038/s41598-025-05218-3

**Published:** 2025-06-20

**Authors:** Taha M. Tiama, Nayera M. El-Sayed, Nabil S. Abdelaziz, Ahmed M. Bayoumy, Hanan Elhaes, Medhat A. Ibrahim

**Affiliations:** 1 Basic Science Department, October High Institute for Engineering and Technology, 6th October City, Cairo Egypt; 2 Physics Department, Faculty of Science, Mansoura University, Mansoura, 35516 Egypt; 3 Biophysics Group, Physics Department, Faculty of Science, Ain Shams University, Cairo, 11566 Egypt; 4 Physics Department, Faculty of Women for Arts, Science and Education, Ain Shams University, Cairo, 11757 Egypt; 5 Spectroscopy Department, National Research Centre, 33 El-Bohouth St., Dokki, 12622 Giza Egypt; 6 Molecular Modeling and Spectroscopy Laboratory, Centre of Excellence for Advanced Science, National Research Centre, 33 El-Bohouth St., Dokki, 12622 Giza Egypt

**Keywords:** Biosensor, Glutamic acid, Graphene oxide, Electronic properties, B3LYP/SDD, DFT, Materials science, Physics

## Abstract

This study investigates the electronic properties of a proposed composite structure consisting of SiO_2_, Pb_3_O_4_, Bi_2_O_3_, and graphene oxide (GO) for glutamic acid (Glu) biosensing applications in aqueous media. Using Density Functional Theory (DFT) at B3LYP functional and SDD basis set, we examine the reactivity and electronic properties of the combination of these structures under weak and complex interaction scenarios with Glu. The study focuses on studying total dipole moments (TDM), HOMO/LUMO bandgaps, molecular electrostatic potential (MEP) maps, reactivity descriptors, and the density of states (DOS) for the proposed model molecules. The calculated TDMs and HOMO/LUMO bandgap energies highlight the highly reactive nature of the 3SiO_2_/GO/Pb_3_O_4_/Bi_2_O_3_ “complex” structure toward the surrounding species. This is because it has the highest TDM (up to 35.1 Debye) and the lowest bandgap energy (decline significantly to 0.158 eV). The MEP maps for the interaction between 3SiO_2_/GO/Pb_3_O_4_/Bi_2_O_3_ and Glu under the two proposed scenarios display markedly different MEP profiles, underscoring the substantial impact of the interaction type. Additionally, the interaction between 3SiO_2_/GO/Pb_3_O_4_/Bi_2_O_3_ “complex” structure and Glu exhibits the highest ionization potential, electron affinity, and electronegativity. The plotted DOS curves of the interaction between the proposed composite structure (both weak and complex forms) and the target analyte reveal that the unoccupied states begin to emerge slightly below − 4.0 eV and − 5.0 eV, then extend towards 0.0 eV, indicating potential excitation energies for electrons. These findings boost the potential of the proposed 3SiO_2_/GO/Pb_3_O_4_/Bi_2_O_3_ structure as a promising candidate for tailoring novel electrode materials for Glu biosensing applications, thereby advancing the development of effective biosensors.

## Introduction

As medical technology advances, there is an increasing demand for faster and more effective diagnostic methods for diseases, including pathogen detection and early diagnosis for cancer and genetic disease. However, traditional diagnostic techniques are often slow, expensive, and require specialized equipment, trained personnel, and complicated facilities, posing significant limitations to wide scale applications^1^. Biosensing technology offers a promising alternative to traditional diagnostic methods by providing precise diagnoses without the logistical constraints commonly associated with conventional techniques^2,3^. Typically, biosensors detect biological signals and convert them into measurable electrical signals, composing of key components, such as bioreceptors, transducers, analytes, and displays^4,5^. A bioreceptor is a biological entity and/or a chemical substance that responds uniquely to the analyte biological target, generating a detectable signal^6^. A transducer provides a specific mechanism that transforms energy from one form to another. Electrochemical sensors with catalytic functions can detect changes in intermediate chemical compounds, classified as either enzymatic or non-enzymatic based on the nature of the catalyst used^7^. Though biosensors based on biological enzymatic entities in the biosensing processes are highly efficient, they suffer from several limitations, including their high sensitivity for environmental conditions (e.g., temperature and pH), in addition to their complexity and high cost which shrink their application in mass scale appliances^8^. Recent research has focused on innovative electrochemical sensors utilizing nanomaterials, which are highly regarded for their superior physical, chemical, optical, and electronic properties compared to bulk materials^9,10^. They mainly rely on nonenzymatic nanomaterial comprising the latest fourth generation of commercial biosensors^11^. They include a broad range of nanomaterials, including metals, metal oxides and carbon-based materials^12–15^.

Metal oxide nanostructures are a key focus in advancing biosensor development due to their nano-sized morphology, strong biocompatibility, non-toxicity, and catalytic capabilities^16,17^. These nanostructured materials also offer enhanced electron transfer kinetics and robust adsorption in various microenvironments^18,19^. Silicon dioxide (SiO_2_), lead (II, IV) oxide (Pb_3_O_4_), and bismuth trioxide (Bi_2_O_3_) are notable metal oxides for biosensing applications due to their unique properties. SiO_2_ offers excellent insulating properties, stability, and biocompatibility, making it an ideal substrate for electrode fabrication^20^. Pb_3_O_4_ and Bi_2_O_3_ are boasted by their enhanced electronic performance, in addition to their semiconducting properties, tunable band nature, and redox activities, enabling efficient charge transfer and high sensitivity in analyte detection processes^21,22^. Likewise, Graphene oxide (GO) has also gained prominence for biosensing applications due to its high electrical conductivity, large surface area, and biological compatibility. It is often utilized to improve the electron transfer kinetics and biomolecule immobilization, boosting the sensitivity and stability of the fabricated biosensors^23,24^. Consequently, we propose that the integration of SiO_2_, Pb_3_O_4_, Bi_2_O_3_, and GO into a single electrode would offer significant potential for advancing biosensor technology. The combination of these materials would provide enhanced electronic and electrochemical properties, ideal for various biosensing applications^25^. This integration supports the development of a highly sensitive and selective biosensor for glutamic acid detection, with potential uses in food quality assessment, biomedical diagnostics, and environmental monitoring.

Glutamic acid (Glu), a crucial neurotransmitter, plays significant roles in the growth, maintenance, reproduction, and immunity processes through its metabolism to α-ketoglutarate and its involvement in the TCA cycle and amino acid biosynthesis^26–28^. It can be synthesized by the body or obtained from dietary sources such as dairy products, poultry, and fish^29^. Deficiencies in glutamic acid can lead to symptoms including anxiety, digestive issues, weakened immunity, and impaired recovery^30^. Consequently, developing a reliable and straightforward method for detecting glutamic acid in biological samples is essential. However, Glu detection is still a challenging process due to its limited distinctive physical properties. Hence, tailoring novel modified electrodes (biosensors) would offer a potential way for rapid and selective detection^31,32^. Therefore, molecular modeling concepts were employed to investigate to what extent the proposed SiO_2_/GO/Pb_3_O_4_/Bi_2_O_3_ structure could perform and operate efficiently in detecting Glu as a biosensor’s active material. Molecular modeling enables researchers to simulate the actions of molecules and materials on an atomic or molecular scale, offering valuable insights into their structure, dynamics, and interactions^33,34^.

Herein, the utilized molecular modeling analysis consists of various steps. The initial step involves modifying graphene oxide with different metal oxides such as SiO_2_, Pb_3_O_4_, and Bi_2_O_3_. Then, the study proceeds to validate the biosensor’s capacity to detect Glu on theoretical and molecular bases. To explore this concept, Density Functional Theory (DFT) at the B3LYP level is conducted to examine the characteristics of the proposed biosensor’s active material (SiO_2_/GO/Pb_3_O_4_/Bi_2_O_3_). Using DFT to compute the molecule’s geometric parameters, atomic charges, and vibrational frequencies provides clearer insight into its electronic properties^35^. The process encompasses sequential functionalization: first GO, then SiO_2_, Pb_3_O_4_, Bi_2_O_3_ (in various interaction configurations), and ultimately, the SiO_2_/GO/Pb_3_O_4_/Bi_2_O_3_-Glu composite. Essential features including total dipole moment, HOMO/LUMO energy gap, molecular electrostatic potential maps and density of states are computed for these model molecules.

## Calculation details

The model molecules investigated in the study underwent computational analysis using the G09 software^36^. This software, running on a personal computer, employed Density Functional Theory (DFT) with the B3LYP^37,38^ functional, and SDD basis set for all calculations. The SDD basis set known for its reliability in providing accurate results for the types of materials studied. Total dipole moment (TDM), HOMO/LUMO energy gap beside molecular electrostatic potential maps were calculated with the same model. Some electronic descriptors such as ionization potential (I), electronic affinity (A), electronic chemical potential (µ), chemical hardness (η), absolute softness (S), and electrophilicity index (ω) were computed based on the values of the calculated HOMO/LUMO energy gap. These descriptors were commonly employed to quantify the reactivity of chemical structures^39^. The band structure is typically determined through density of states (DOS) diagrams^40^. The DOS plots were generated using DFT calculations at B3LYP level with the SDD basis set. The computational software employed for these calculations was Gauss Sum 3, which is widely recognized for its accuracy and reliability in electronic structure analysis. The procedure involved optimizing the geometry of each model molecule to its ground state before calculating the electronic structure parameters to obtain the eigenvalues. Finally, the DOS plots are generated by projecting these eigenvalues onto a suitable energy range to visualize the density of electronic states. The self-consistent field (SCF) energy was converged to within an energy tolerance of 10^−6^ eV. This ensures that the total energy calculations reach a sufficiently accurate and stable solution. For geometry optimization, the convergence criteria for the maximum force were set to 10^−4^ eV/Å. Additionally, the maximum displacement allowed for atomic positions was constrained to 10^−3^ Å to achieve accurate geometrical configurations.

## Results and discussions

### Building model molecules

**Fig. 1:**
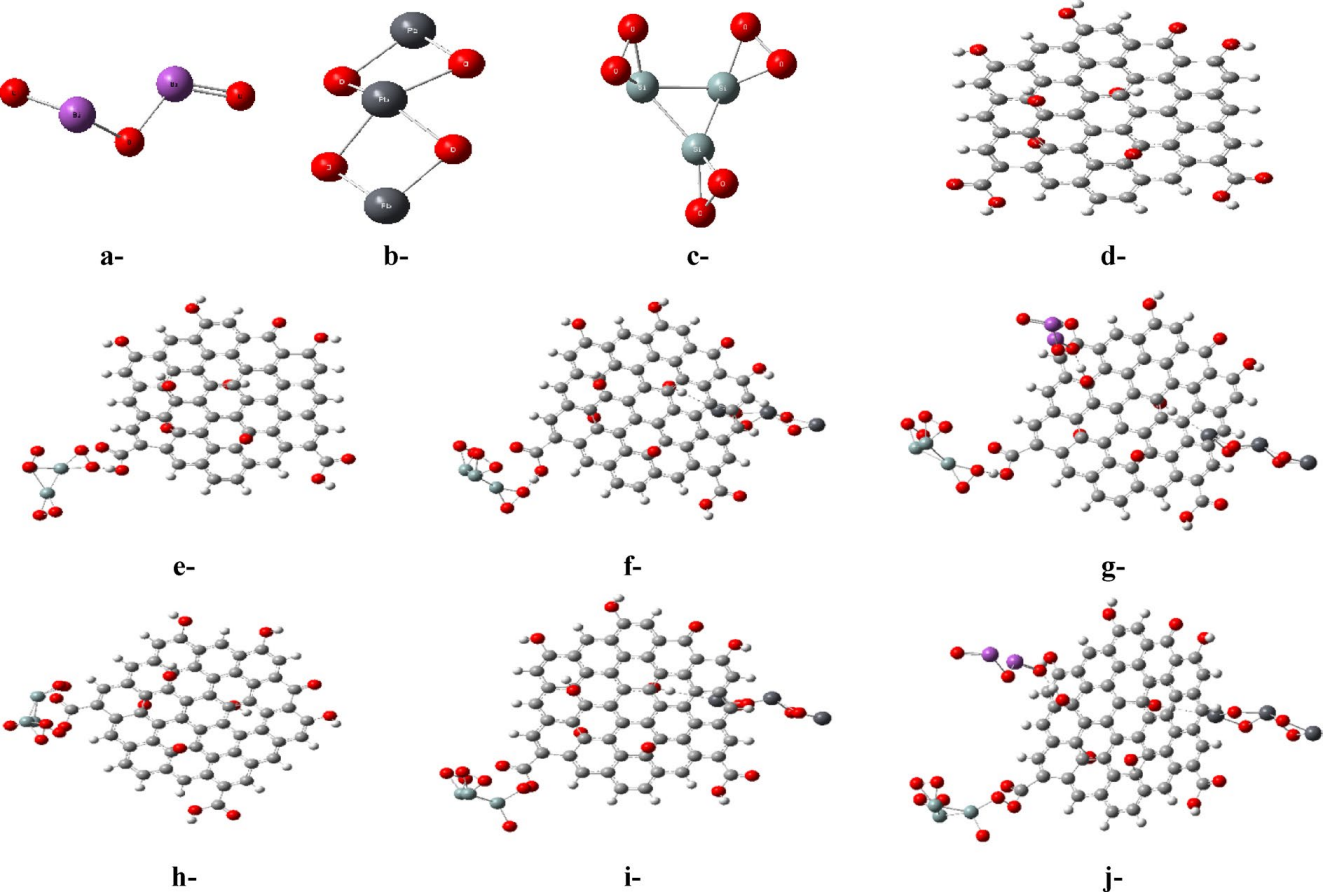
Model molecules studied: a-Bi_2_O_3_, b-Pb_3_O_4_, c-3SiO_2_, d-GO, e-3SiO_2_/GO (weak interaction), f-3SiO_2_/GO/Pb_3_O_4_ (weak interaction), g-3SiO_2_/GO/Pb_3_O_4_/Bi_2_O_3_ (weak interaction), h-3SiO_2_/GO (complex interaction), i-3SiO_2_/GO/Pb_3_O_4_ (complex interaction), j-3SiO_2_/GO/Pb_3_O_4_/Bi_2_O_3_ (complex interaction).

The model molecules examined were constructed and described in detail, as illustrated in Fig. 1. Specifically, the model molecules appear in the following sequence: Fig. 1a represents Bi_2_O_3_, Fig. 1b corresponds to Pb_3_O_4_, Fig. 1c represents 3SiO_2_, Fig. 1d stands for graphene oxide (GO), Fig. 1e depicts 3SiO_2_/GO via “weak interaction”, Fig. 1f illustrates 3SiO_2_/GO/Pb_3_O_4_ via “weak interaction”, Fig. 1g shows 3SiO_2_/GO/Pb_3_O_4_/Bi_2_O_3_ via “weak interaction”, Fig. 1h represents 3SiO_2_/GO via “complex interaction”, Fig. 1i corresponds to 3SiO_2_/GO/Pb_3_O_4_ via “complex interaction”, and Fig. 1j depicts 3SiO_2_/GO/Pb_3_O_4_/Bi_2_O_3_ via “complex interaction”.

This section presents the composition of the proposed electrode’s active material for the detection of glutamic acid in a sequential manner to mimic the experimental steps in the electrode manufacturing process. To further elaborate, the model starts with three units of SiO_2_ to play the role of biocompatible substrate due to its excellent insulating properties and unique stability required for electrodes’ accurate performance. Then, GO is virtually cast over the SiO_2_ substrate to represent the electrode’s major component due to its highly promising features for biosensing applications, including outstanding electrical conductivity, large specific surface area, and compatibility with biological systems. Finally, the electrode composition is further enhanced via integration of both Pb_3_O_4_ and Bi_2_O_3_ due to their tunable electronic band structures and redox activities which could boost the efficient and swift charge transfer processes and facilitate the detection of target analytes with high sensitivity and selectivity.

This work presents the two major schemes of interactions between chemical structures. In other words, weak and complex interaction schemes are proposed between the suggested candidates. The “weak interaction” represents the formation of a physical hydrogen bonding between the selected active sites. On the same manner, the “complex interaction” represents the formation of a covalent bond through a hidden esterification process.

### Calculated physical parameters

The constructed structures received calculations using the B3LYP method and the SDD basis set within the framework of DFT theory. Various physical and electronic parameters, including total dipole moment (TDM) and HOMO/LUMO bandgap energies (ΔE) were obtained from these calculations and are presented in Table 1.

**Table 1:** B3LYP/SDD calculated total dipole moment (TDM) as Debye and HOMO/LUMO energy gap (ΔE) as eV.

Structure	TDM, Debye	ΔE, eV
1-Bi_2_O_3_	4.092	3.132
2-Pb_3_O_4_	2.548	1.908
3-3SiO_2_	5.639	1.841
4-GO	4.056	1.111
5-3SiO_2_/GO “weak interaction”	21.838	0.319
6-3SiO_2_/GO/Pb_3_O_4_ “weak interaction”	14.713	0.201
7-3SiO_2_/GO/Pb_3_O_4_/Bi_2_O_3_ “weak interaction”	21.601	0.158
8-3SiO_2_/GO “complex interaction”	26.184	0.485
9-3SiO_2_/GO/Pb_3_O_4_ “complex interaction”	29.858	0.260
10-3SiO_2_/GO/Pb_3_O_4_/Bi_2_O_3_ “complex interaction”	35.063	0.248

The total dipole moment (TDM) serves as a critical indicator of the chemical reactivity of the substances under investigation^41^. It assesses their ability to interact with neighboring entities based on their predominant charges. Table 1 presents the calculated TDM values for the studied structures. It is worth noting that the resulting TDM values range from 2.5 Debye for Pb_3_O_4_ to 5.6 Debye for the constructed three units of SiO_2_ (3SiO_2_). Notably, both Bi_2_O_3_ and GO structures exhibit similar TDM values, indicating comparable chemical reactivity. However, TDM alone does not capture the full scope of reactivity, which also involves other factors such as electronic structure, surface interactions, and catalytic properties. Beyond TDM, the electronic properties of these materials differ significantly. For instance, GO shows a much smaller HOMO/LUMO energy gap (ΔE = 1.111 eV) compared to Bi_2_O_3_ (ΔE = 3.132 eV). This indicates potentially higher electronic conductivity and more active electronic behavior, which could contribute to its different reactivity profile compared to Bi_2_O_3_. The interaction between GO and 3SiO_2_ significantly influences the resulting structures’ TDM, whether via weak or complex interactions, resulting in higher TDM values of 21.8 and 26.2 Debye, respectively, indicating highly reactive structures, especially in the complex form. However, the addition of Pb_3_O_4_ to 3SiO_2_/GO has a notable negative impact on lowering the TDM in the weak form to 14.7 Debye. Conversely, the addition of Pb_3_O_4_ to 3SiO_2_/GO in the complex form plays a positive role in increasing TDM from 26.2 to 29.9 Debye. Similarly, the interaction of Bi_2_O_3_ with the proposed 3SiO_2_/GO/Pb_3_O_4_ structure results in highly reactive structures in both weak and complex forms. In the context of composite structures like 3SiO_2_/GO/Pb_3_O_4_/Bi_2_O_3_, the combined effects of each component can lead to enhanced overall reactivity. The significantly higher TDM observed in the composite structures (e.g., 35.063 Debye for the “complex interaction” scenario) reflects the synergistic effects of integrating multiple materials, which often results in improved reactivity compared to the individual components. It is noteworthy that the complex form appears to be more effective in yielding reactive structures. This suggests that the proposed 3SiO_2_/GO/Pb_3_O_4_/Bi_2_O_3_ structure exhibits high reactivity toward surrounding species, making it a potential candidate for biosensing applications.

**Fig. 2:**
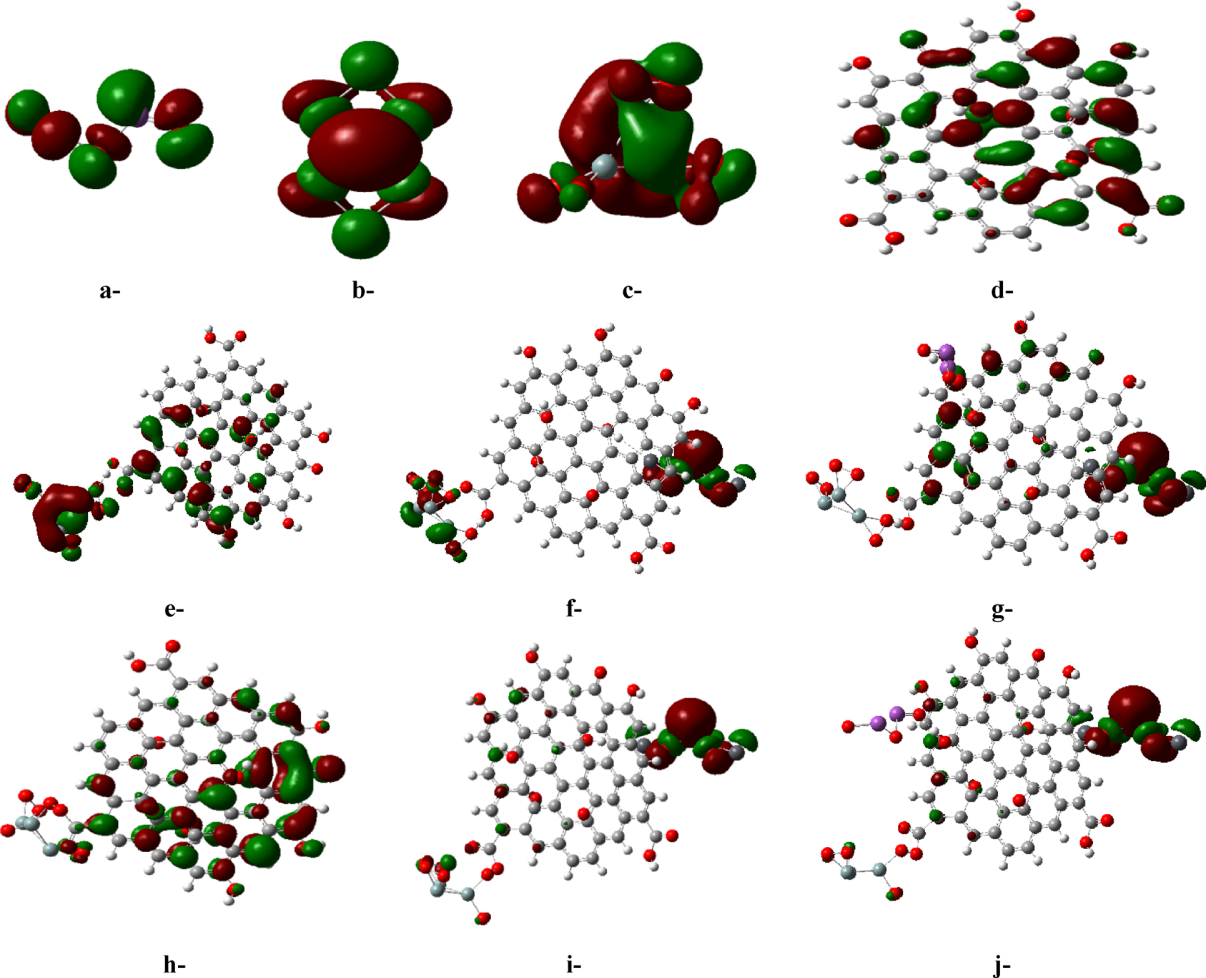
Iso-surfaces for HOMO (highest occupied molecular orbital) and LUMO (lowest unoccupied molecular orbital) of the studied model molecules calculated using the B3LYP/SDD functional: a-Bi_2_O_3_, b-Pb_3_O_4_, c-3SiO_2_, d-GO, e-3SiO_2_/GO (weak interaction), f-3SiO_2_/GO/Pb_3_O_4_ (weak interaction), g-3SiO_2_/GO/Pb_3_O_4_/Bi_2_O_3_ (weak interaction), h-3SiO_2_/GO (complex interaction), i-3SiO_2_/GO/Pb_3_O_4_ (complex interaction), and j-3SiO_2_/GO/Pb_3_O_4_/Bi_2_O_3_ (complex interaction) (HOMO: red, LUMO: green).

Like TDM, the HOMO/LUMO bandgap energies of the proposed candidates were examined as potential electronic parameters. These energies are commonly used to describe the ease of electron transfer from the valence to the conduction band in a chemical entity^42^. As stated before, GO exhibits the lowest bandgap energy of 1.1 eV compared to the other three candidates (Bi_2_O_3_, Pb_3_O_4_, and 3SiO_2_), which have energies of 3.1, 1.9, and 1.8 eV, correspondingly. On the same manner of TDM, all suggested structures (whether in weak or complex forms) have significantly lower bandgaps compared to their individual components, indicating highly conductive structures that provide efficient pathways for streaming charge carriers. For instance, the addition of both Pb_3_O_4_ and Bi_2_O_3_ to the 3SiO_2_/GO structure positively impacts the reduction of its bandgap from 0.319 eV to 0.201 and 0.158 eV in the weak form, and from 0.485 eV to 0.26 and 0.248 eV in the complex states, respectively. Therefore, the bandgap energies listed in Table 1 are consistent with the previously discussed TDM values, indicating highly reactive and conductive structures suitable for sensing applications, revealing the synergistic impact of integrating both Pb_3_O_4_ and Bi_2_O_3_ to tune its electronic features and justifying the order of building this model molecule. Moreover, Fig. 2 illustrates the visualized distribution of the B3LYP/SDD calculated HOMO/LUMO for the studied model molecules. It demonstrates a uniform distribution of the molecular orbitals in all selected candidates. However, the interaction of GO with 3SiO_2_, Pb_3_O_4_, and Bi_2_O_3_ significantly allows the tuning of the HOMO and LUMO distribution in the resulting structures. This suggests reactive chemical entities, confirming the positive impact of the addition of the proposed molecules on reducing the bandgap energies, indicative of highly conductive structures.

### Molecular electrostatic potential (MEP) maps

Moreover, MEP maps, color-coded maps commonly generated to visualize the charge distribution patterns and active sites of the structures under investigation^43,44^, are derived from the analysis of electron density distribution around nuclei^45,46^. These maps serve to illustrate a chemical structure’s capability to facilitate the streaming of charge carriers^47^. Like other map types, MEP maps consist of colored profiles that distinguish various active sites and charge values^43^. Typically, MEP maps feature a spectrum of colors ranging from red, representing highly negative regions, to dark blue, indicating highly positive ones^48,49^. It is important to note that the highly electronegative elements are depicted in red, while the less electronegative ones are colored blue, and green signifies neutral sites^50^.

**Fig. 3:**
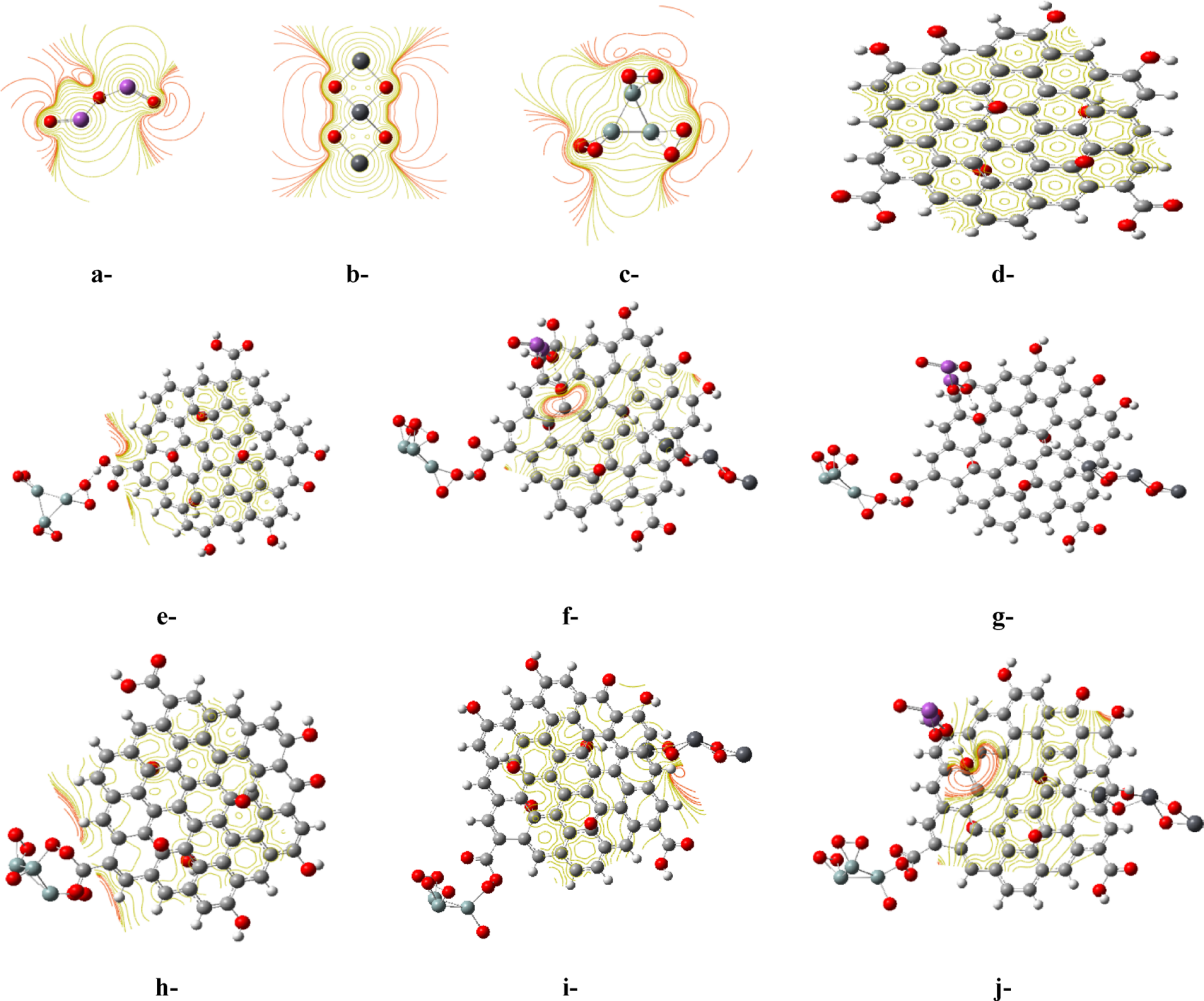
B3LYP/SDD calculated molecular electrostatic potential (MEP) for the studied model molecules: a-Bi_2_O_3_, b-Pb_3_O_4_, c-3SiO_2_, d-GO, e-3SiO_2_/GO (weak interaction), f-3SiO_2_/GO/Pb_3_O_4_ (weak interaction), g-3SiO_2_/GO/Pb_3_O_4_/Bi_2_O_3_ (weak interaction), h-3SiO_2_/GO (complex interaction), i-3SiO_2_/GO/Pb_3_O_4_ (complex interaction), and j-3SiO_2_/GO/Pb_3_O_4_/Bi_2_O_3_ (complex interaction).

Furthermore, MEP analysis can provide further insights into the types of chemical addition reactions most likely to occur at the various active sites. For instance, nucleophilic additions are expected to occur predominantly at red sites, while electrophilic additions are mainly associated with blue regions^51^. Figure 3 displays the molecular electrostatic potential (MEP) maps for the model molecules, calculated using the B3LYP/SDD functional. The maps are presented at the same density functional theory (DFT) level to provide a comparative view of the electrostatic properties across the different structures under investigation. Generally, the four candidates exhibit red lines surrounding the highly electronegative oxygen atoms, indicating nucleophilic active sites, while other elements such as carbon, hydrogen, lead, and bismuth appear in greenish yellow, representing neutral moieties. However, combining these structures tends to result in nearly neutral structures, except for some sites containing oxygenated species, which appear in red.

Table 2 presents the B3LYP/SDD calculated reactivity descriptors for the studied structures, such as ionization potential (I), electron affinity (A), electronic chemical potential (μ), chemical hardness (η), absolute softness (S), and electrophilicity index (ω). Regarding the ionization potential ($I=-{E}_{HOMO}$), it signifies the energy needed to remove an electron from the highest occupied molecular orbital (HOMO) to surpass its binding energy with the nucleus of a chemical structure. GO exhibits the lowest ionization potential among the four individuals, measuring only 4.996 eV in an agreement with the previously presented HOMO/LUMO bandgap energy. The integration of 3SiO_2_ to GO, either through weak or complex interactions, results in an increase in its ionization potential to 6.006 and 5.633 eV, correspondingly, revealing much more stable structure. However, the interaction of GO/3SiO_2_ with Pb_3_O_4_ and Bi_2_O_3_ slightly lowers its ionization potential, enhancing their electrical properties. On contrary, the electronic affinity ($A=-{E}_{LUMO}$) reflects a material’s ability to accept electrons into its lowest unoccupied molecular orbital (LUMO) from neighboring entities. Similar to ionization potential, GO possesses the smallest electron affinity at 3.88 eV compared to the other structures. The interaction of GO with other species enhances the capability of the resulting structures to accept more electrons. Therefore, electronic chemical potential (µ), or electronegativity, represents an average value of both ionization potential and electronic affinity. It dictates the direction of charge transfer between adjacent chemical structures. According to the HSAB theory, charges typically transfer from a substance of lower electronegativity to one of higher electronegativity until reaching equilibrium^52^. It is important to note that, by convention, the electronic chemical potential (μ) is expressed as a negative value and is related to electronegativity (χ) by the relationship μ = − χ. Thus, while μ is negative, electronegativity is positive. The three units of SiO_2_ exhibit the highest electronegativity of 6.96 eV, while GO has the lowest value of 4.44 eV. Incorporating SiO_2_, Pb_3_O_4_, and Bi_2_O_3_ into GO increases its electronegativity, whether in weak or complex forms. Furthermore, hardness ($\eta$) and softness ($S$) indicate the resistance and ease with which a chemical structure can donate its electrons to neighboring entities. Bi_2_O_3_ demonstrates the lowest degree of softness of 0.64 (eV)^−1^. Conversely, the proposed structure, in weak or complex forms, exhibits the highest softness of 12.63 and 8.08 (eV)^−1^, respectively. Lastly, the electrophilicity index ($\omega =\frac{{\mu }^{2}}{2\eta }$) is a crucial parameter for assessing the capability of a chemical structure to attract external electron density from a nucleophilic one. The three units of SiO₂ exhibit the highest electrophilicity index of 26.34 eV compared to the other individual components. It is worth noting that the exceptionally high electrophilicity index (ω) value of 164.514 eV observed for the 3SiO₂/GO/Pb₃O₄/Bi₂O₃ “weak interaction” system is attributed to its exceptionally low chemical hardness (η) value of 0.079 eV. This low η significantly amplifies the ω value, despite the chemical potential (μ) remaining within the range of the other systems. Such a low chemical hardness indicates that the system is highly reactive and has a strong tendency to accept electrons. While these elevated ω values may suggest chemically excessive reactivity, they reflect the intrinsic electronic characteristics of this particular composite structure. These findings highlight that the proposed structures possess high reactivity and excellent potential for electrical and electronic applications, given their enhanced capacity to facilitate the flow of charge carriers.

**Table 2:** B3LYP/SDD calculated reactivity descriptors for the studied structures.

Structure	I (eV)	A (eV)	μ (eV)	η (eV)	S (eV)^−1^	ω (eV)
1- Bi_2_O_3_	7.144	4.012	5.578	1.566	0.639	9.935
2- Pb_3_O_4_	6.637	4.729	5.683	0.954	1.048	16.930
3- 3SiO_2_	7.884	6.043	6.964	0.921	1.086	26.339
4- GO	4.996	3.884	4.440	0.556	1.800	17.740
5-3SiO_2_/GO “weak interaction”	6.006	4.655	5.330	0.676	1.480	21.030
6-3SiO_2_/GO/Pb_3_O_4_ “weak interaction”	5.821	4.747	5.284	0.537	1.862	25.991
7-3SiO_2_/GO/Pb_3_O_4_/Bi_2_O_3_ “weak interaction”	5.184	5.025	5.104	0.079	12.629	164.514
8-3SiO_2_/GO “complex interaction”	5.633	5.148	5.391	0.243	4.120	59.859
9-3SiO_2_/GO/Pb_3_O_4_ “complex interaction”	5.559	5.299	5.429	0.130	7.680	113.175
10-3SiO_2_/GO/Pb_3_O_4_/Bi_2_O_3_ “complex interaction”	5.373	5.125	5.249	0.124	8.077	111.270

**Fig. 4:**
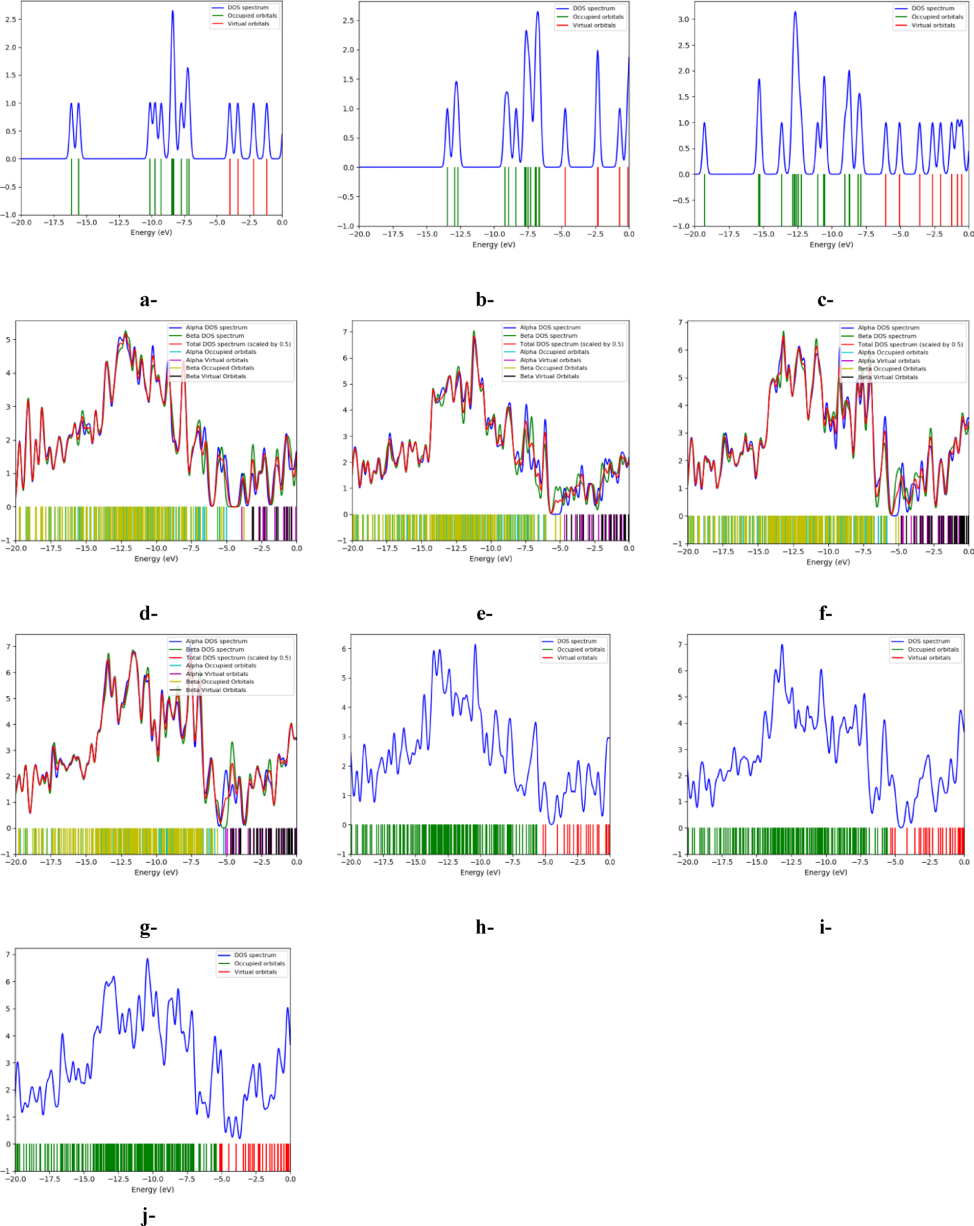
B3LYP/SDD calculated density of states (DOS) for the studied model molecules: a-Bi_2_O_3_, b-Pb_3_O_4_, c-3SiO_2_, d-GO, e-3SiO_2_/GO (weak interaction), f-3SiO_2_/GO/Pb_3_O_4_ (weak interaction), g-3SiO_2_/GO/Pb_3_O_4_/Bi_2_O_3_ (weak interaction), h-3SiO_2_/GO (complex interaction), i-3SiO_2_/GO/Pb_3_O_4_ (complex interaction), j-3SiO_2_/GO/ Pb_3_O_4_/Bi_2_O_3_ (complex interaction).

Figure 4 presents the density of states (DOS) profiles of the investigated model systems, offering comprehensive insights into their electronic structures and energy levels distribution^53^. Each DOS curve reflects the electronic configuration derived from the molecular orbital calculations, whose total number contributes to characterizing the electronic behavior and reactivity of the system, as previously reported in literature on small biomolecules^54^. In the presented DOS plots, the green vertical lines represent the occupied molecular orbitals; those filled with electrons in the ground state. These are found below the Fermi level and define the stable electronic structure of the molecular system. Conversely, the red lines denote the unoccupied (virtual) orbitals, which lie above the Fermi level and correspond to energy levels accessible upon electronic excitation. The blue DOS curve reflects the density of the available electronic states at each energy level, and its peaks signify the energies at which the electrons are most densely populated or likely to be excited. The distinct separation between the occupied and unoccupied levels defines the bandgap; a critical parameter that influences the molecule’s electrical conductivity, optical properties, and chemical reactivity. To further elaborate, a wider bandgap reflects a stable, less reactive system with lower polarizability, whereas a narrower bandgap implies a higher electronic polarizability and potential chemical softness, suggesting higher chemical reactivity boosting the possibility of redox activities. This concept is consistent with what was stated earlier at^54^ for the interpretation of the bandgap modulation of ascorbic acid to solvent interaction and model selection.

For the investigated systems, their DOS profiles reveal that they share the same general features indicating that their electronic structure are preserved across the different suggested models, since they all composed of the same building blocks, such as SiO₂, GO, Pb₃O₄, and Bi₂O₃ moieties. However, differences in the intensity and distribution of DOS peaks reflect variations in molecular interactions and structural complexity. These variations suggest that the local environment around the active sites and the electronic coupling between the proposed components can modulate the electronic density and contribute to different bandgap energies. Similar to findings by Nasidi et al.^54^, the DOS plots reveal that the common components share the same general electronic features; however, the differences in peak intensities and distributions reflect variations in their corresponding molecular interactions.

The final proposed configurations, featuring either weak or complex interactions—namely 3SiO₂/GO/Pb₃O₄/Bi₂O₃—were further examined as active materials for biosensing glutamic acid (Glu). Two interaction scenarios between Glu and the proposed composite were studied: weak and complex binding. Table 3 presents the computed physical and electronic parameters, such as total dipole moments (TDM) and bandgap energies for these structures, highlighting key electronic changes induced by Glu adsorption. For instance, total dipole moment (TDM) is highly sensitive to molecular interactions and changes in electronic distribution due to the surrounding chemical environment. To further clarify, larger TDM values indicate stronger polarization effects and increased charge asymmetric distribution caused by the analyte (e.g., Glu) adsorption, boosting the electrostatic interactions between them. This charge redistribution significantly improves the interaction strength, thereby increasing the sensor’s sensitivity and ability to detect Glu via measurable changes in the electronic environment. In this study, the proposed weak interaction system exhibits a TDM of 63.9 Debye, while the complex one leads to an even higher TDM of 73.0 Debye, demonstrating a notable enhancement in the polarity and chemical reactivity upon Glu adsorption. These elevated TDM values correlate with stronger donor–acceptor interactions and enhanced charge delocalization, which are essential for effective biosensing performance.

**Table 3:** B3LYP/SDD calculated TDM as Debye and HOMO/LUMO energy gap (ΔE) as eV for the two proposed glutamic sensing schemes.

Structure	TDM, Debye	ΔE, eV
1. 3SiO_2_/GO/ Pb_3_O_4_/Bi_2_O_3_/Glu “weak interaction”	63.858	0.166
2. 3SiO_2_/GO/ Pb_3_O_4_/Bi_2_O_3_/Glu “complex interaction”	73.006	0.447

Likewise, bandgap energy plays a significant role in influencing the electrical conductivity and electron transfer properties of sensors’ active materials. A narrow HOMO/LUMO bandgap allows easier electron excitation, thereby enhancing the electron transfer process which is vital for the sensor’s performance and analyte detection. This reduced bandgap energy leads to increased electrical conductivity, enabling faster and more efficient charge transfer when Glu molecules interact with the composite surface. Such efficient charge transfer is essential for generating strong and rapid sensor signals, directly improving biosensor sensitivity and signal response. The calculations show that the bandgap energies of the weak and complex interactions are 0.166 and 0.447 eV, respectively, and both are slightly larger than the pristine material. This rise suggests a moderate modification in the electronic configuration of the proposed composite material in response to Glu adsorption, where the complex interaction causes more pronounced changes. Despite this increase, the bandgaps remain sufficiently small to support effective electron transport, balancing sensor stability and responsiveness. Together, the enhanced total dipole moments and tuned bandgap energies reflect the influence of Glu binding on the electronic properties of the suggested composite. The increased polarity (high TDM) enhances the sensor’s sensitivity by amplifying electrical signal changes upon Glu adsorption. Meanwhile, the bandgap variations modulate the sensor’s conductivity, affecting the speed and efficiency of charge transfer processes which are essential for the signal generation step. Thus, the correlation between TDM and bandgap values provides a comprehensive insight into the sensing mechanism, revealing that the interaction-induced changes in the electronic environment improve both the sensitivity and selectivity of the 3SiO₂/GO/Pb₃O₄/Bi₂O₃ biosensor for glutamic acid detection. These findings support the material’s potential as an effective electrode for aqueous Glu sensing applications.

It is worth noting that the TDM of the proposed complex interaction is much higher than that of the weak one, which can be rationalized on the physical and chemical levels by considering several contributing factors. For instance, the active material (3SiO_2_/GO/ Pb_3_O_4_/Bi_2_O_3_) built upon complex interactions between its components are most likely to have much stronger electron donor–acceptor behaviors between the highly electronegative elements and the lower ones, creating a semipermanent electron delocalization, arising partial negative and positive charges on its constituents. This is clearly obvious in the values of TDM of pristine 3SiO_2_/GO/ Pb_3_O_4_/Bi_2_O_3_ prior to Glu binding which are 21.601 and 35.063 Debye for the weak and strong interactions, correspondingly (see Table 1). The impact of this enhanced electron density redistribution profile extends upon the Glu adsorption, rising the TDM of the complex configuration relative to the weak one. Moreover, such electron configurations may facilitate the enhanced orbital overlap between the lone pairs on oxygen or nitrogen atoms of Glu and the vacant orbitals of metal oxides of the biosensor’s active material, which would lead to the formation of coordinate bonds or boost the proposed charge-assisted hydrogen bonding. This overlap increases the extent of electronic coupling, contributing to charge delocalization over the interacting system. As a result, electron density is displaced over a broader spatial region, increasing the molecular dipole moment. This delocalized charge distribution manifests as a larger total dipole moment, which directly impacts the sensor’s ability to create an electrical signal in response to Glu adsorption.

**Fig. 5:**
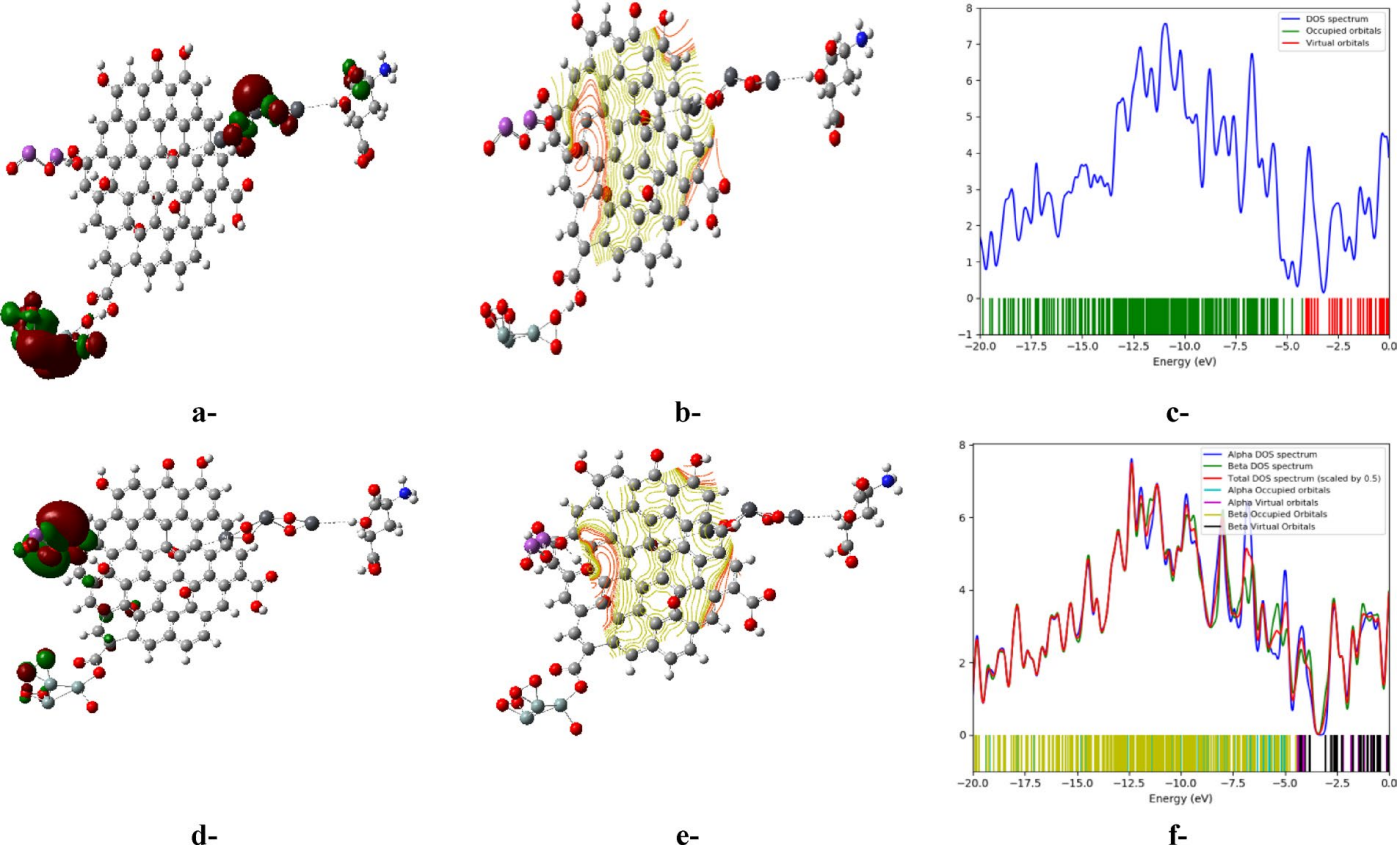
B3LYP/SDD calculated: a-HOMO/LUMO, b-MEP, and c-DOS for the studied model molecule 3SiO_2_/GO/Pb_3_O_4_/Bi_2_O_3_/Glu (weak interaction), and d-HOMO/LUMO, e-MEP, and f-DOS for the studied model molecule 3SiO_2_/GO/Pb_3_O_4_/Bi_2_O_3_/Glu (complex interaction).

Moreover, Fig. 5 illustrates the distribution of HOMO and LUMO for the proposed weak and complex structures, depicted as Fig. 5a and d, respectively. In Fig. 5a, it is evident that the HOMO and LUMO are concentrated around both 3SiO_2_ and Pb_3_O_4_ near the interaction site with glutamic acid. Conversely, Fig. 5d displays their distribution around all interaction sites between GO and Bi_2_O_3_, as well as 3SiO_2_. These distinct distribution patterns highlight the significant impact of the interaction type, whether weak or complex. Furthermore, Fig. 5b and e elucidates the MEP maps for the two proposed scenarios, showcasing a consistent pattern with the HOMO/LUMO distributions. The two proposed structures exhibit notably different MEP profiles, emphasizing the significant effect of the interaction type. Moreover, the density of states (DOS) for the two scenarios is plotted to further illustrate their electronic properties. Figure 5c and f suggests that in the case of weak and complex interactions, the unoccupied states (indicated by red lines) begin to emerge slightly below − 4.0 eV and − 5.0 eV, respectively, in the 3SiO_2_/GO/Pb_3_O_4_/Bi_2_O_3_/Glu system. These states then extend towards 0 eV, indicating potential excitation energies for electrons. In conclusion, these findings confirm that the suggested 3SiO_2_/GO/Pb_3_O_4_/Bi_2_O_3_ structure holds as a promising candidate for manufacturing electrodes designed for detecting glutamic acid in aqueous environments.

Furthermore, Table 4 presents several computed physical reactivity parameters, including ionization potential (I), electron affinity (A), electronic chemical potential (μ), chemical hardness (η), absolute softness (S), and electrophilicity index (ω). It is important to note that, by convention, the electronic chemical potential (μ) is expressed as a negative value and is related to electronegativity (χ) by the relationship μ = –χ. Thus, while μ is negative, electronegativity is positive. From Table 4, it is evident that the interaction between 3SiO₂/GO/Pb₃O₄/Bi₂O₃ and glutamic acid through complex formation exhibits the highest ionization potential, electron affinity, and electronegativity, with values of 4.9 eV, 4.4 eV, and 4.7 eV, respectively (electronegativity obtained as − μ), compared to 4.2 eV, 4.1 eV, and 4.1 eV for the weak interaction. However, the weak interaction provides a more accessible pathway for electron donation, as indicated by a higher softness value of 12.02 (eV)^−1^ relative to only 4.5 (eV)^−1^ for the complex form. Similarly, the weak interaction results in a significantly higher electrophilicity index of 103.3 eV, compared to 48.7 eV for the complex pattern.

**Table 4:** B3LYP/SDD calculated reactivity descriptors for the studied structure.

Structure	I (eV)	A (eV)	μ (eV)	η (eV)	S (eV)^−1^	ω (eV)
1-3SiO_2_/GO/ Pb_3_O_4_/Bi_2_O_3_/Glu “weak interaction”	4.227	4.061	− 4.144	0.083	12.029	103.269
2-3SiO_2_/GO/Pb_3_O_4_/Bi_2_O_3_/Glu “complex interaction”	4.893	4.445	− 4.669	0.224	4.465	48.669

### Stability of the studied compounds

**Fig. 6:**
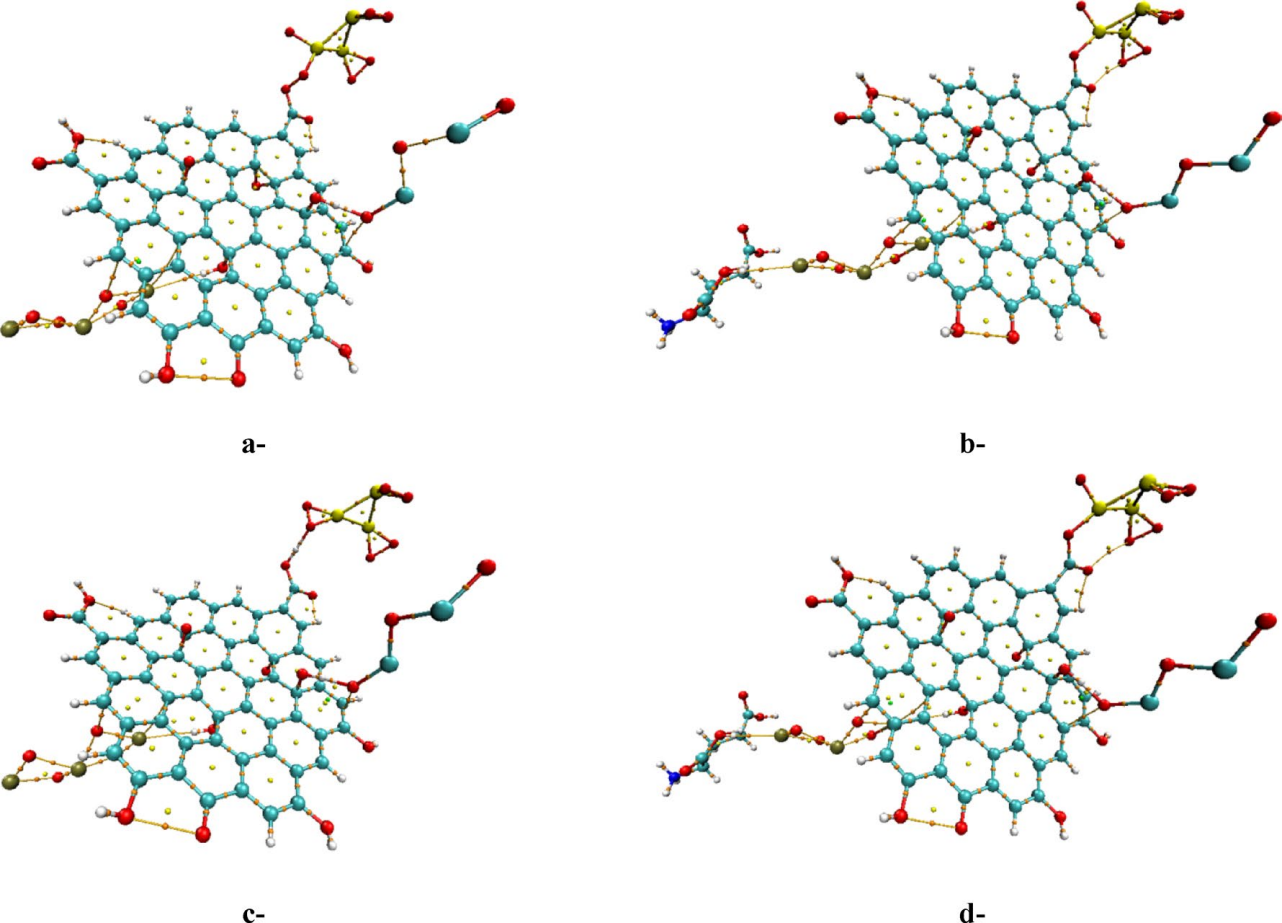
B3LYP/SDD calculated QTAIM for the studied model molecules: a-SiO_2_/GO/Pb_3_O_4_ (weak interaction), b-3SiO_2_/GO/Pb_3_O_4_/Bi_2_O_3_/Glu (weak interaction), c-3SiO_2_/GO/Pb_3_O_4_/Bi_2_O_3_/Glu (complex interaction) and d-3SiO_2_/GO/ Pb_3_O_4_/Bi_2_O_3_ (complex interaction).

The structures studied were subjected to Quantum Theory of Atoms in Molecules (QTAIM) calculations to assess their stability. QTAIM is a well-established theoretical framework that provides detailed insights into the electronic density distribution by identifying bond paths and bond critical points (BCPs), which are essential for understanding bonding interactions and adsorption processes on surfaces^55^. In QTAIM analysis, the electron density (ρ) at BCPs between interacting atoms serves as an indicator of bond strength, where higher ρ values typically correspond to stronger bonding interactions. Importantly, the nature of the bond can be inferred from the values of the Laplacian function of the electron density, ∇^2^ρ(r), and the total electron energy density, H(r), at these points. When both ∇^2^ρ(r) and H(r) are negative, this indicates a region of high charge population characteristic of covalent (shared-shell) bonding interactions. In contrast, positive values for both parameters are indicative of closed-shell interactions, such as weak hydrogen bonds, van der Waals forces, or electrostatic interactions, which are non-covalent in nature. As shown in Fig. 6, the QTAIM analysis reveals that the studied structures primarily exhibit non-covalent interactions consistent with weak bonding characteristics. However, the simultaneous negativity of ∇^2^ρ(r) and H(r) at certain BCPs confirms the presence of covalent bonding interactions within the composite system. These covalent bonds contribute significantly to structural stability. Furthermore, after incorporating Bi₂O₃/Glu, additional hydrogen bonding interactions were observed, further enhancing the overall stability through synergistic effects of both strong covalent and supplementary weak non-covalent forces. Thus, the QTAIM results affirm that the composite maintains its integrity and stability due to a combination of covalent bonds confirmed by the negative ∇^2^ρ(r) and H(r) values, along with stabilizing non-covalent interactions.

## Conclusion

In conclusion, a comprehensive investigation of a proposed composite 3SiO_2_/GO/Pb_3_O_4_/Bi_2_O_3_ structure for the detection of glutamic acid for biosensing applications has been proposed. Through computational analysis, various physical parameters have been examined, shedding light on the reactivity and electronic properties of the structure in both weak and complex interaction scenarios. The analysis of reactivity physical parameters provides valuable insights into the ionization potential, electron affinity, and electronegativity of the structures. The resulting data indicate that the TDM values varied significantly among the proposed structures. For instance, Pb_3_O_4_ has the lowest TDM of 2.548 Debye, whereas 3SiO_2_ has the highest one of 5.639 Debye. Consequently, the suggested composite structures show notably higher TDM values, indicating enhanced reactivity. For instance, the complex interaction of 3SiO_2_/GO/Pb_3_O_4_/Bi_2_O_3_ exhibits a TDM of 35.063 Debye. The bandgap energy (ΔE) of GO is the lowest at 1.111 eV, suggesting higher electronic conductivity. However, the incorporation of Pb_3_O_4_ and Bi_2_O_3_ into the 3SiO_2_/GO structure decline the bandgap significantly. For example, the weak interaction form of 3SiO_2_/GO/Pb_3_O_4_/Bi_2_O_3_ has a bandgap of 0.158 eV. Like the energy gap, GO has the lowest ionization potential (I) at 4.996 eV, indicating that it easily loses electrons. The composite structure 3SiO_2_/GO/Pb_3_O_4_/Bi_2_O_3_ in weak interaction form shows an exceptionally high electrophilicity index (ω) of 164.514, due to its low chemical hardness (η) of 0.079 eV.

The MEP maps illustrate the charge distribution and active sites. Oxygen atoms appear highly electronegative (red regions), indicating nucleophilic active sites. DOS plots confirm the complex electronic structure of the proposed materials, showing a distinct separation between the occupied (green) and the unoccupied (red) states, indicating its potential for various electronic applications. The 3SiO_2_/GO/Pb_3_O_4_/Bi_2_O_3_ structure, particularly the complex form, demonstrates high reactivity and electronic conductivity, making it a promising candidate for biosensing applications. The interaction of this composite with glutamic acid is further explored, revealing highly reactive entities with TDM values of 63.858 Debye for the weak interaction and 73.006 Debye for the complex interaction. The bandgap energies for these interactions are 0.166 eV and 0.447 eV, respectively, indicating suitable electronic properties for the sensor applications. Overall, these results support the assumption that the 3SiO_2_/GO/Pb_3_O_4_/Bi_2_O_3_ structure holds great potential as a promising candidate for tailoring the fabrication process of electrodes for the sensitive detection of glutamic acid in aqueous media.

## Data Availability

The data that support the findings of this study are available from the corresponding author upon reasonable request.
